# Effects of Apparent Temperature on the Incidence of Ventricular Tachyarrhythmias in Patients With an Implantable Cardioverter–Defibrillator: Differential Association Between Patients With and Without Electrical Storm

**DOI:** 10.3389/fmed.2020.624343

**Published:** 2021-01-15

**Authors:** Hui-Chun Huang, Pei-Chin Suen, Jih-Shin Liu, Cheryl Chia-Hui Chen, Yen-Bin Liu, Chu-Chih Chen

**Affiliations:** ^1^Division of Cardiology, Department of Internal Medicine, National Taiwan University Hospital, National Taiwan University College of Medicine, Taipei, Taiwan; ^2^Department of Nursing, College of Medicine, National Taiwan University, National Taiwan University Hospital, Taipei, Taiwan; ^3^Division of Biostatistics and Bioinformatics, Institute of Population Health Sciences, National Health Research Institutes, Zhunan, Taiwan; ^4^Research Center for Environmental Medicine, Kaohsiung Medical University, Kaohsiung, Taiwan

**Keywords:** electrical storm, implantable cardioverter-defibrillator, incidence rate, relative humidity, ventricular tachyarrhythmia

## Abstract

**Background:** Electrical storm (ES) has profound psychological effects and is associated with a higher mortality in patients with implantable cardioverter–defibrillator (ICD). Assessing the incidence and features of ES, is vital. Previous studies have shown winter peaks for ventricular tachyarrhythmia (VTA) in ICD patients. However, the effects of heat with a high relative humidity remain unclear. Thus, this study aimed to assess the nonlinear and lagged effects of apparent temperature [or heat index (HI)] on VTA among patients with and without ES after ICD implantation.

**Methods:** Of 626 consecutive patients who had ICDs implanted from January 2004 to June 2017 at our hospital, 172 who experienced sustained VTAs in ICD recording were analyzed, and their clinical records were abstracted to assess the association between VTA incidence and HI by time-stratified case-crossover analysis. Cubic splines were used for the nonlinear effect of HI, with adjustment for air pollutant concentrations.

**Results:** A significant seasonal effect for ES patients was noted. Apparent temperature, but not ambient temperature, was associated with VTA occurrences. The low and high HI thresholds for VTA incidence were <15° and >30°C, respectively, with a percentage change in odds ratios of 1.06 and 0.37, respectively, per 1°C. Lagged effects could only be demonstrated in ES patients, which lasted longer for low HI (in the next 4 days) than high HI (in the next 1 day).

**Conclusion:** VTA occurrence in ICD patients was strongly associated with low HI and moderately associated with high HI. Lagged effects of HI on VTA were noted in patients with ES. Furthermore, patients with ES were more vulnerable to heat stress than those without ES. Patients with ICD implantation, particularly in those with ES, should avoid exposure to low and high HI to reduce the risk of VTAs, improve quality of life and possibly reduce mortality.

## Introduction

Ventricular tachyarrhythmias (VTAs) are associated with a greater risk of sudden death. Treatment with implantable cardioverter–defibrillators (ICDs) has become the standard intervention for patients at risk of life-threatening VTAs, including ventricular tachycardia (VT) and ventricular fibrillation (VF) (1). ICDs also provide detailed information about VTAs, which could help evaluate the nature and distribution of ventricular arrhythmias. Electrical storm (ES), which is defined as three or more separate VT/VF episodes leading to ICD therapy within 24 h, not only produces profound psychological morbidity but is also associated with increased mortality (2). Hence, clinical assessment of the incidence and features of ES is vital; however, ES is unpredictable (3). Studies in the United States (4), Germany (5), Switzerland (6), and Canada (7) found that VTA incidence peaks in winter. In some studies in Japan (8) and Korea (9), the peak incidence of ICD shocks occurs in spring and early summer in patients with Brugada syndrome. High temperature is also reported to cause sudden cardiac deaths and cardiovascular mortalities (10, 11).

Relative humidity (RH) in the aforementioned study areas of continental climate is generally low compared with that in subtropical or tropical areas, such as Southeast Asian countries. The climate in Taiwan, which is an island located in the western Pacific Ocean, is generally warm and humid (with a year-round RH of approximately 76%). Previous researches showed that there is a significant interaction effect of temperature and RH on cardiovascular mortality (12, 13). High RH inhibits body heat dissipation when ambient temperature is high and insulation when temperature is low; both augment the sensation of temperature. Thus, apparent temperature or heat index (HI), which is a composite index of ambient temperature and RH that could determine how the body perceives temperature, has been proposed to have a more eminent cardiovascular effect than ambient temperature alone (13). Hence, this study aimed to assess the nonlinear and delayed association between sustained VTAs and HI in patients with ICD implantation (both with and without ES) and quantify the risk of VTAs due to apparent temperature.

## Materials and Methods

### Data

This retrospective cohort study included 626 consecutive patients who had ICD implantation at National Taiwan University Hospital from January 1, 2004, to June 30, 2017. Data on sex, age at index date, smoking status, place of residence, and medical history, including hypertension, diabetes mellitus, hyperlipidemia, coronary artery disease, congestive heart failure, medication use, clinical diagnosis for defibrillator implantation, and initial left ventricular ejection fraction (LVEF), were extracted from medical records. The setting of ICD therapy for each patient, including VT zone, VF zone, antitachycardia pacing (ATP) therapy, and shock energy, was determined by the physician. This study was approved by the National Taiwan University Hospital Research Ethics Committee.

### Ventricular Tachyarrhythmia Events

**Figure 1:**
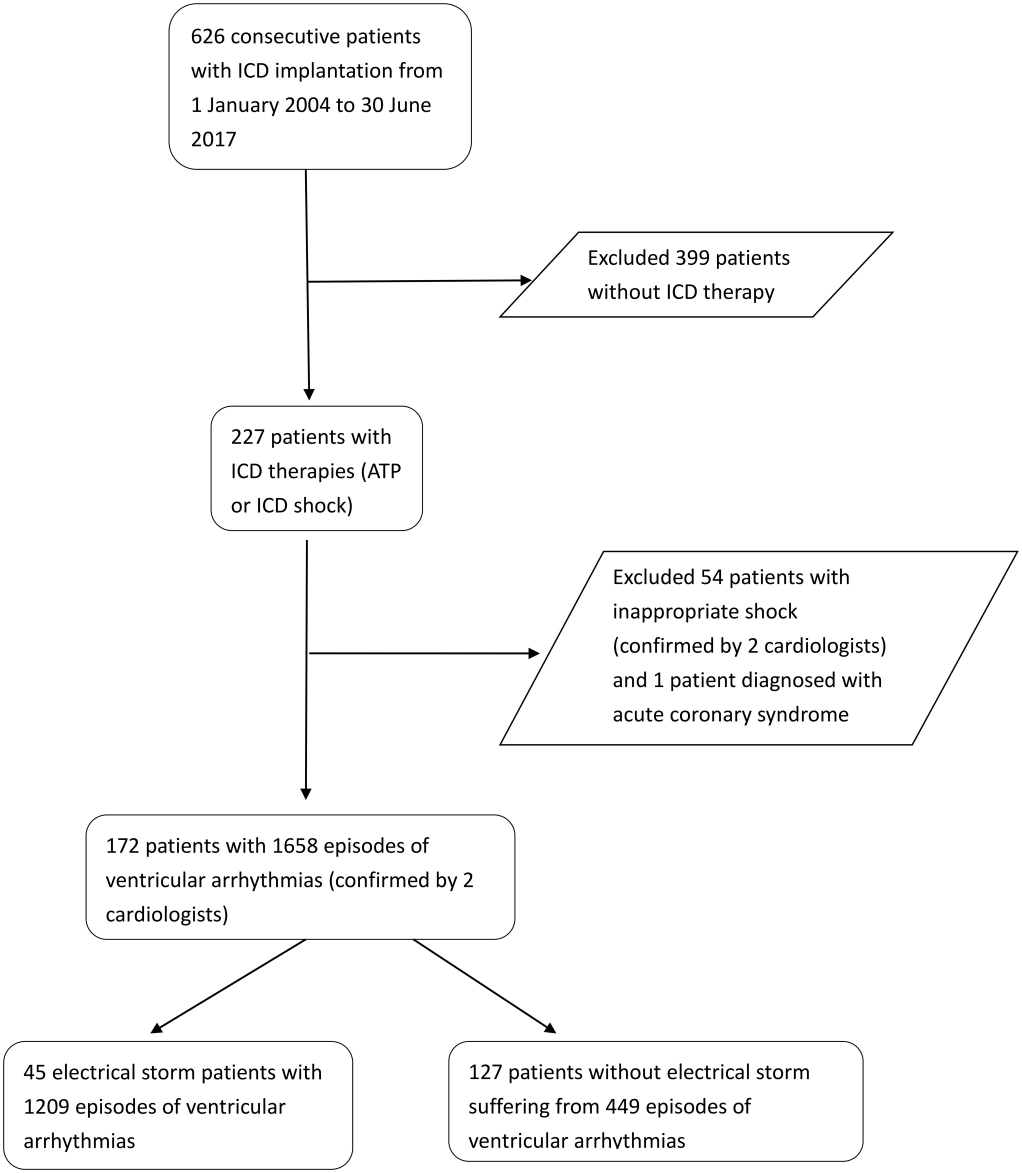
Flowchart of patient selection.

Information on each VTA event, including the date, timing, and duration of each arrhythmia, was extracted from the ICD device during the outpatient follow-up period. Two independent cardiologists reviewed the records to discriminate VTAs from supraventricular arrhythmia and determined whether the therapy was appropriate shock or inappropriate shock. Patients with inappropriate ICD shocks, including supraventricular tachycardias, double counting of R waves, oversensing of T waves as R waves, and an artifact or noise, and those with VTAs who experienced acute coronary syndrome were excluded in our study. We considered VTA that recurred within 5 min as the same episode as the preceding event regardless of the total number of ATP or shock therapy (2). Figure 1 shows the flowchart of patient selection. Forty-five patients with ICD therapies experiencing ES and 127 patients with isolated appropriate ICD therapies were included in the final analysis.

### Air Quality Data

We obtained hourly measurements of air pollutant concentrations (PM_2.5_, PM_10_, NO, NO_2_, SO_2_, O_3_, and CO) from the air-monitoring stations of Taiwan Environmental Protection Agency from January 1, 2004, to June 30, 2017 and estimated the 24-h average concentrations at the patients' residence. Geographic information system was employed to determine the coordinates of the home address. We divided the residence of the patients into seven geographical areas across Taiwan for their spatial heterogeneity: North, Northern Central (NC), Central (C), Central South (CS), South (S), Northern East (NE), and East (E).

A generalized additive model for each area was applied to estimate the air pollutant exposures based on the measures from the monitoring stations. The model included three meteorological covariates (daily 24-h average temperature, wind speed, and RH) that influence pollutant dispersion. Township population density and traffic load (total counts of automobiles and motorbikes) near the monitoring station were also included as land use covariables in the model (14). We used cubic splines to model the meteorological covariables, population density, and traffic load and validated the model using a cross-validation technique (15). For patients residing <1 km away from a monitoring station, the estimates of air pollutant exposures were obtained directly from the measures of that station. An ordinary kriging technique was applied to the model residuals for the spatially smoothed estimates across the study areas of western Taiwan (N, NC, C, CS, and S), which were the main residential areas with geospatial homogeneity.

### Heat Index Data

We calculated the HI (°F) based on Steadman's formula or Rothfusz's full formula, depending on whether the calculated HI was </≥80°F, with adjustments according to different ranges of RH and temperature (16) (see Supplementary Material, for the detailed formula). The calculated HI in °F was converted to °C. We obtained the 24-h average temperature and RH from the air-monitoring station that is closest to the patients' residence.

### Statistical Analysis

We used a bidirectional time-stratified case-crossover approach to assess the association between the incidence of sustained ventricular arrhythmia and HI. This design allowed for investigation of short-term environmental exposures, such as air pollution and temperature, by comparing the subject's exposure before the time of the event (case period) with the exposures at separate control periods. A conditional logistic regression was used to analyze the subject's event onset and the matched sets (control periods) for the differences of exposure status. Thus, each patient serves as his or her own control and adjusts for confounding by season, month, day of the week, time trend upon exposure, and individual characteristics. The case periods were the days of sustained VTA occurrences in patients with ICD. For each case period, the matched control periods (days) were the same weekdays of the same month. For preliminary analysis, we used a conditional logistic regression model to assess the statistical association between the incidence of sustained VTA and the composite effects of ambient temperature and RH. We included composite weather condition based on temperature and RH (numbered 1–6) as a covariable; we categorized ambient temperature into low, medium, and high and RH into low and high. The cutoff values were determined using Youden's index based on the corresponding receiver operating characteristic curve. Other covariables adjusted in the model included estimated 24-h air pollutant concentrations and an indicator variable of whether an episode occurred on the previous day. Subsequently, we assessed the association between the incidence of ICD therapy and HI using cubic splines with three degrees of freedom to account for the nonlinear effect of low and high HI, after adjusting for air pollutant concentrations and previous-day incident.

To assess monthly and seasonal effects, we employed a generalized linear model with a logit link for recurrent VTA incidents of each patient during the follow-up period. Variations among the patients were treated as random effects. To assess the lagged effects of HI on ICD therapies, we fitted the conditional logistic models with different lag structures for the calculated HIs from the current day (lag 0) up to the sixth lagged day (lag 6). Moreover, we performed subgroup analysis by evaluating the differential effects of HI on VTAs between (1) ES group and non-ES group; (2) ischemic cardiovascular disease (CVD) group and non-ischemic CVD group. Because most of the patients were admitted for the management of ES. Therefore, some of the VTA episodes had occurred in the hospital where the temperature and humidity were well controlled. A sensitivity analysis was performed by excluding the patients who experienced in-hospital ES episodes. We calculated the relative risks (RRs) as the ratios of the corresponding estimated probabilities. The analyses were performed using the SAS software (version 9.4) (SAS Institute Inc., Cary, NC) and the R software (version 3.5.1) for statistical computing. A test statistics with a *P* value < 0.05 was considered statistically significant.

## Results

### Study Population and Meteorological Data

Between January 1, 2004, and June 30, 2017, 1,659 ICD therapies (ATP or shock) were recorded in 172 patients with ICD during a median follow-up of 65 months. Mean age was 55.5 ± 18.0 years and mean LVEF was 49.7 (18.5%). The major causes of ICD implantation were ischemic cardiomyopathy (*n* = 64, 37.2%) and dilated cardiomyopathy (*n* = 44, 25.5%). Among the 172 patients with ICD therapy, 45 (26.2%) experienced ES, 69.2% used beta-blocker, and 77.3% received amiodarone (Table 1).

**Table 1:** Clinical characteristics of ICD patients with confirmed sustained ventricular tachyarrhythmias.

	**ICD patients with VTAs**
	***N* = 172**
Age, mean (SD), years	55.6 (17.8)
Male (%)	133 (76.9)
Hypertension (%)	87 (50.3)
Diabetes mellitus (%)	41 (23.7)
Dyslipidemia (%)	43 (24.9)
Smoking (%)	30 (17.3)
CAD (%)	79 (45.7)
CHF (%)	78 (45.1)
CVA (%)	17 (9.8)
**Diagnosis at ICD implantation (%)**	
Ischemic cardiomyopathy	65 (37.6)
Dilated cardiomyopathy	44 (25.4)
Hypertrophic cardiomyopathy	10 (5.8)
Arrhythmogenic right valve dysplasia	10 (5.8)
Long QT syndrome	7 (4.0)
Brugada syndrome	13 (7.5)
Congenital heart disease	5 (2.9)
Idiopathic	19 (11.0)
**Indication of ICD implantation (%)**	
Primary prevention	20 (11.6)
Secondary prevention	153 (88.4)
**Ejection fraction (%)**	
≧50	85 (49.4)
40–49	26 (15.0)
<40	62 (36.0)
**Concomitant medication (%)**
β-blockers	119 (68.8)
Amiodarone	133 (76.9)
ACEi/ARB	86 (50.0)

*ACEi/ARB, angiotensin-converting enzyme inhibitor/angiotensin II receptor blocker; CAD, coronary artery disease; CHF, congestive heart failure; CVA, cerebrovascular accident; ICD, implantable cardioverter–defibrillators; VTAs, ventricular tachyarrhythmias*.

During the study period, the lowest and highest recorded 24-h average temperature was 7.7 and 33.6°C, respectively, with a median of 24.0°C, and the lowest and highest RH was 24.5 and 97.7%, respectively, with a median of 74.5%. The calculated 24-h average HI ranged from 6.7 to 41.4°C, with a median of 24.6°C. The estimated PM_2.5_ (PM_10_) concentration ranged from 4.2 (9.1) μ*g*/*m*^3^ to 107.4 (587.5) μ*g*/*m*^3^, with an interquartile range of 9.1 (20.1) μ*g*/*m*^3^. Table 2 lists the exposure distributions of HI, temperature, RH, and estimated air pollutant concentrations for the 172 patients.

**Table 2:** Distribution of the estimated air pollutant concentrations and weather data from January 1, 2004, to June 30, 2017.

**Measure**	**No.of days**	**Min**	**5%**	**25%**	**50%**	**75%**	**95%**	**Max**
Heat index (°C)	3,424	6.73	13.22	18.79	24.57	31.87	36.32	41.40
Temperature (°C)	3,424	7.72	13.75	18.88	24.04	28.38	30.83	34.05
Relative humidity (%)	3,424	24.54	58.61	68.00	74.46	81.04	88.92	97.70
PM_2.5_ (μg/m^3^)	3,424	4.17	13.63	19.44	23.97	30.41	44.75	107.38
PM_10_ (μg/m^3^)	3,434	9.08	25.19	33.17	40.25	53.25	82.34	587.51
CO (ppm)	3,439	0.13	0.30	0.46	0.57	0.72	1.04	2.50
NO_2_ (ppb)	3,432	1.96	10.36	16.93	20.65	24.74	32.10	54.54
O_3_ (ppb)	3,397	6.14	16.06	20.56	24.62	29.64	39.45	81.20
SO_2_ (ppb)	3,428	0.56	2.22	2.96	3.65	4.54	5.66	22.44

*CO, carbon monoxide; NO_2_, nitrogen dioxide; O_3_, ozone; PM, particulate matter; SO_2_, sulfur dioxide*.

### Monthly and Seasonal Pattern of Ventricular Tachyarrhythmia Events

**Figure 2:**
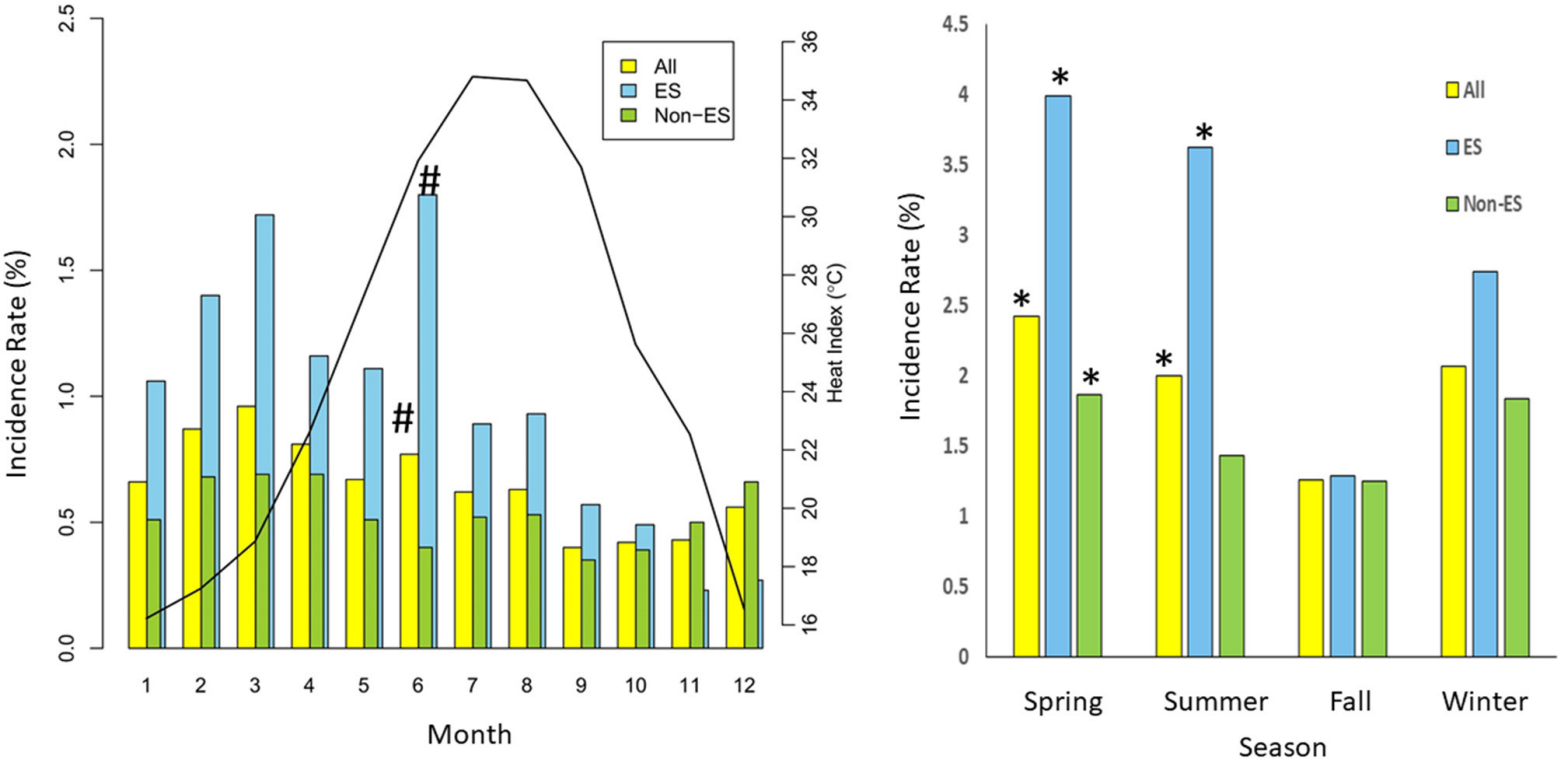
Annual distribution of VTA incidence rate (per person-month) (vertical bar) of 172 patients with ICD (yellow), 45 patients with electrical storm (ES) (blue), and 127 non-ES patients (green). Solid line indicates mean monthly heat index between January 1, 2004, and June 30, 2017. *means *P* < 0.05 as compared with fall; # means *P* < 0.05 as compared with reference month.

Figure 2 shows the monthly incidence rates (IRs) for all, ES, and non-ES patients. The monthly overall IR of sustained VTA was highest in March (0.96% per person-month) and lowest in September (0.40% per person-month). The monthly IRs for patients with ES were generally much higher than those for non-ES patients, with the highest (1.80%) and lowest (0.23%) IRs noted in June and November, respectively. By contrast, the monthly difference in IRs for patients without ES were non-significant. As shown in Figure 2, sustained VTA occurred more frequently in spring and summer than in fall. A significant seasonal effect on VTA occurrence was observed in patients with ES in spring and summer; for non-ES patients, a significant effect was noted in spring.

### Nonlinear and Delayed Associations Between HI and the ICD Therapy Incidence

**Figure 3:**
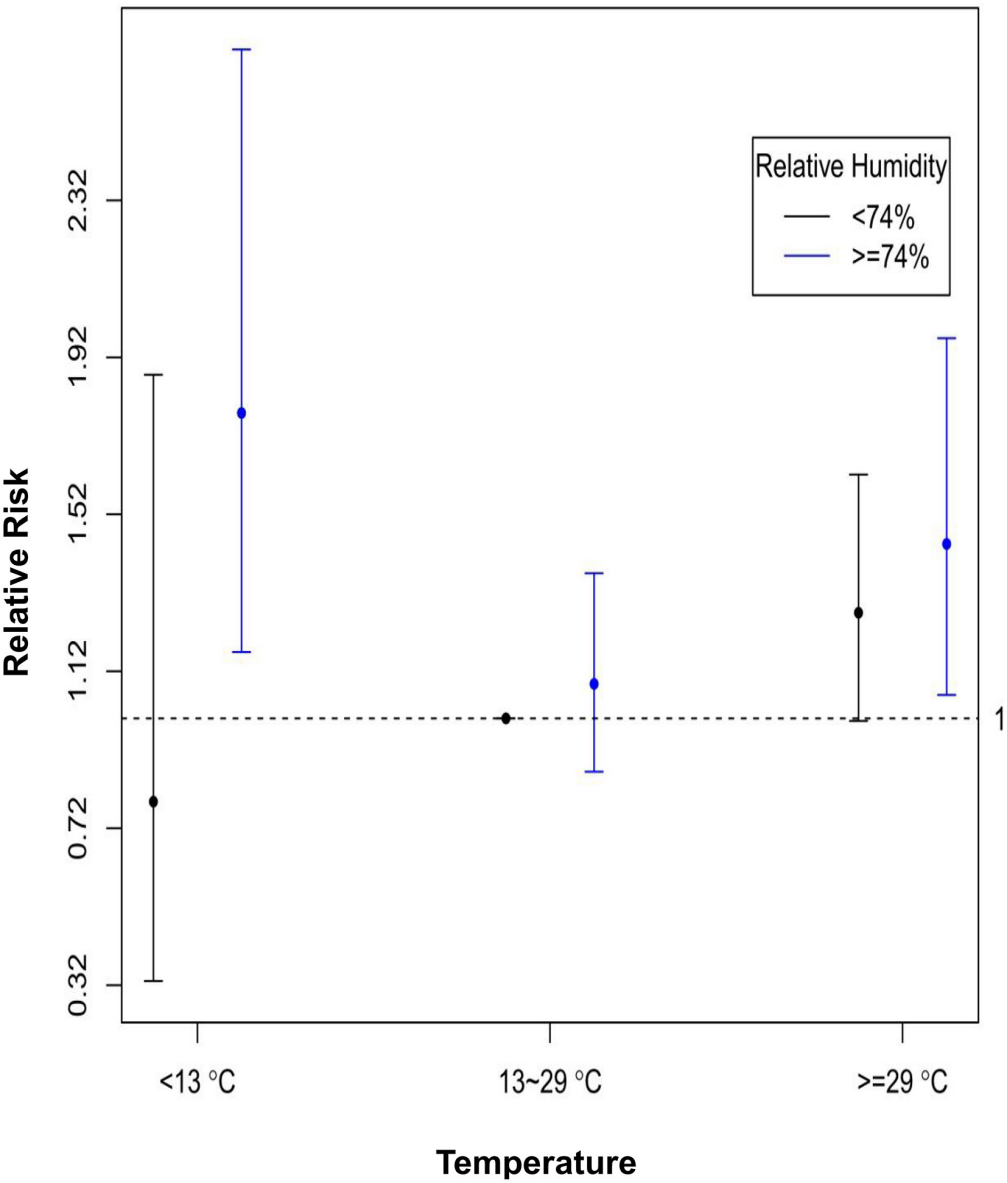
Relative risks of VT incidence of composite conditions of temperature and relative humidity, with modest temperature (13–29°C) and low relative humidity (<74%) as the reference. The dots are the relative risks, and the bars are the corresponding 95% confidence intervals.

For the association between the incidence of ICD therapy and the composite effect of temperature and RH, Figure 3 shows that high RH (≥74%) had a significant increase in RR (i.e., >1) of VA in low (<13°C) and high (≥29°C) temperatures as compared with modest temperature (13–29°C) and low RH (<74%). These results suggested that RH could modify the relationship between temperate and VTA occurrence. Thus, we chose a HI (an index that combines temperature and RH) as an alternative indicator of ambient temperature and used a nonlinear model to analyze the combined effects of temperature and RH on VTA incidents.

**Figure 4:**
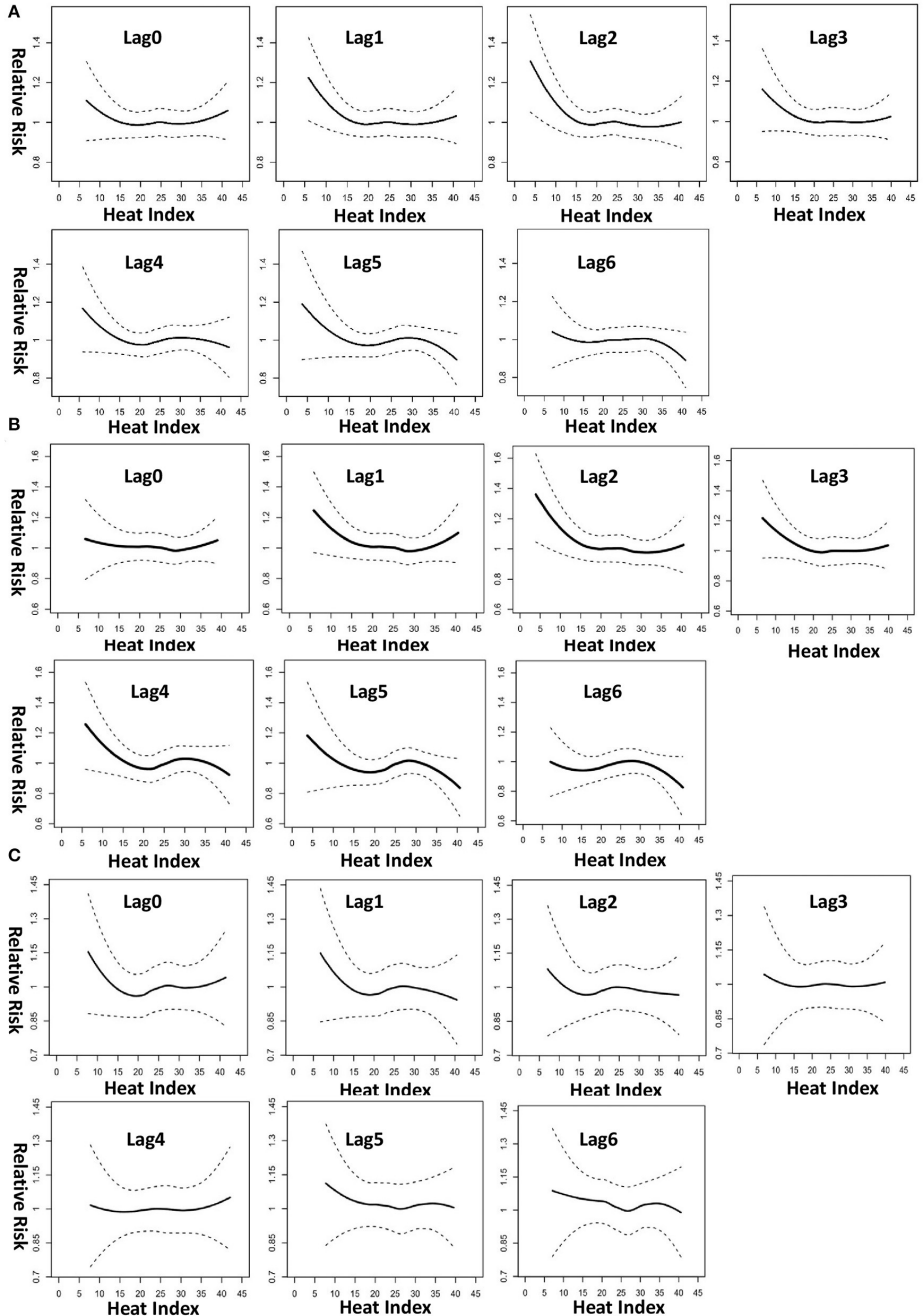
Lag-specific relative risk of VT incidence at different heat indices, with reference at 24.5°C, for **(A)** all 172 patients, **(B)** 45 patients with ES, and **(C)** 127 non-ES patients. The plots were generated using locally weighted scatterplot regression from the output of conditional logistic regression model with cubic spline effect for heat index.

**Figure 5:**
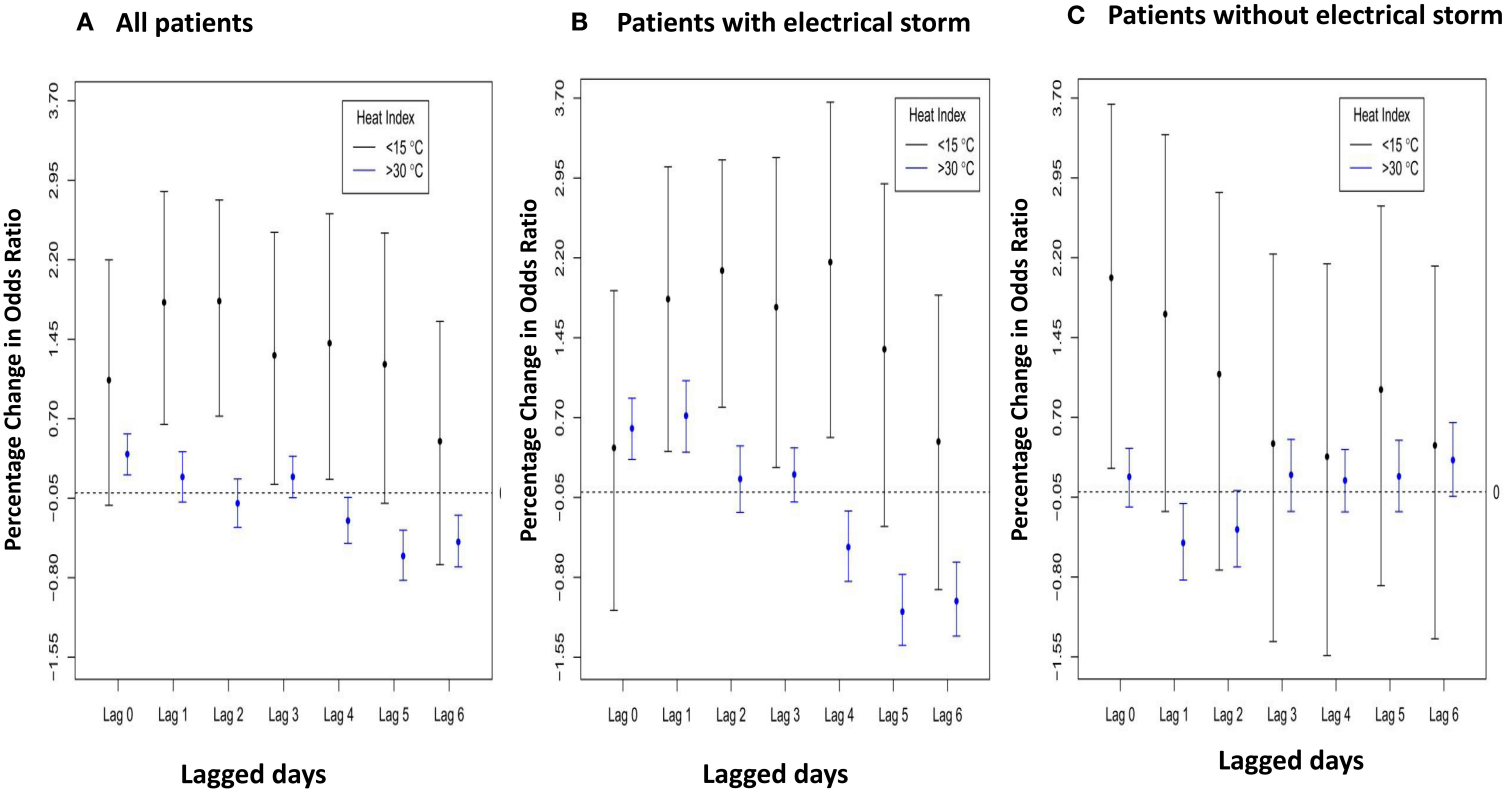
Lag-specific percentage change in odds ratio per 1°C decrease (increase) when daily HI < 15°C (>30°C) for **(A)** all 172 patients, **(B)** 45 patients with ES, and **(C)** 127 non-ES patients.

**Figure 6:**
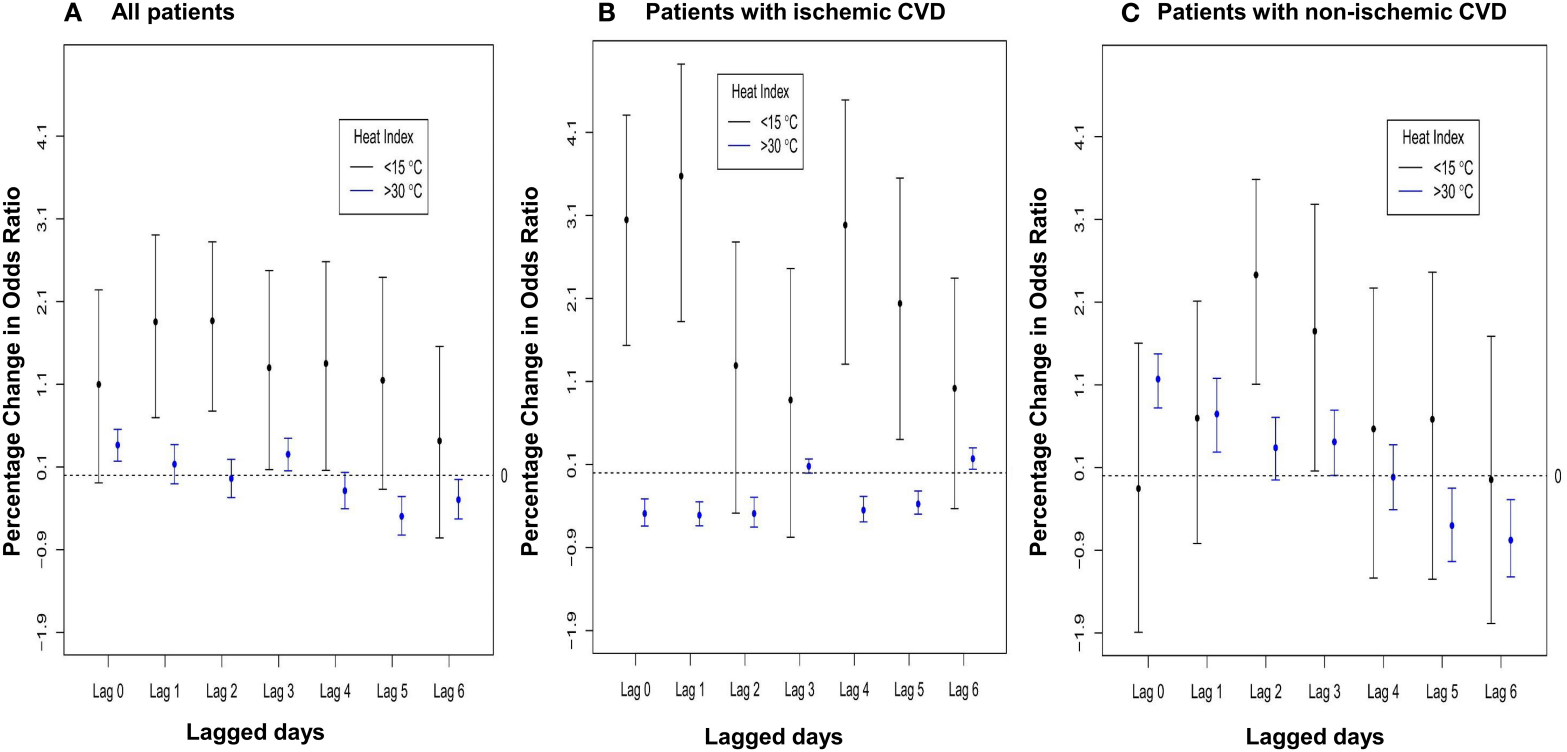
Lag-specific percentage change in odds ratio per 1°C decrease (increase) when daily HI < 15°C (>30°C) for **(A)** all 172 patients, **(B)** patients with ischemic CVD and **(C)** patients with non-ischemic CVD.

Figure 4 shows the nonlinear and lag-specific RR of VTA incidence at different HIs from 0 to 6 days, with a reference HI at 24.5°C. RR increased with either higher or lower HI, with thresholds at 15°C for low HI and 30°C for high HI (lag 0). In patients with ES, the lag effects of low HI were long-lasting (in the next 4 days). However, for high HI, modest lag effects (in the next day only) were noted in patients with ES (Figure 4B), whereas the lag effect for non-ES patients was null (Figure 4C). Moreover, Figure 5 shows the lag-specific percentage change in odds ratio (OR) for a 1°C decrease in HI at 15°C or increase at 30°C. In all patients, the OR increased by 1.06% (95% CI −0.12 to 2.20%) for a 1°C decrease at 15°C and 0.37% (95% CI 0.17–0.56%) for a 1°C increase at 30°C (lag 0) (Figure 5A). The percentage changes of OR in different lags were different between patients with ES and those without ES. In patients with ES, the percentage changes below 15°C were significantly >0 at lag 1 to lag 4, while the percentage changes above 30°C were also significantly >0 at lag 1 (Figure 5B). However, the percentage changes of OR for below 15°C and above 30°C were both non-significant at all lags in non-ES patients (Figure 5C). The acute and lagged effects of low and high HI on VTA incidents in patients with ES and those without ES are summarized in Table 3. We also found there is differential effects of HI on VTAs in different etiology of CVD. The effects of low HI on VTAs seemed more prominent in patients with ischemic CVD than those with nonischemic CVD. The percentage changes of VTA occurrences below 15°C were significantly larger at lag 0 to lag 1. Meanwhile, the nonlinear and delay effects of high HI on VTAs can only be demonstrated in patients with nonischemic CVD. These results are shown in Figure 6.

**Table 3:** The acute and lagged effects of low and high HI on VTA incidents in patients with ES and those without ES.

	**Association**	**All ICD**	**Patients with ES**	**Non-ES**
		***N* = 172**	***N* = 45**	***N* = 127**
Cold effect	Acute	lag 0 (±)	lag 0 (–)	lag 0 (+)
(HI < 15*C*)	Delay	lag 1–4 (+)	lag 1–4 (+)	lag 1–4 (–)
Heat effect	Acute	lag 0 (+)	lag 0 (+)	lag 0 (–)
(HI > 30*C*)	Delay	lag 1 (–)	lag 1 (+)	lag 1–6 (–)

*+ (–): significant (non-significant) association between VTAs and HI*.

Besides, patients who had a VTA occurrence on the previous day had a high OR (6.80, 95% CI 4.97–9.30), which was consistent with the lag effects of HI on VTAs. Air pollutant PM_2.5_ did not have a significant association with sustained VTA incidence (OR 0.993, 95% CI 0.982–1.004). Similarly, all the other air pollutants had no significant association with VTA incidence and had similar outcomes when adjusted in the model (data not shown). The sensitivity analysis showed the occurrence of VTAs were still significantly associated with both low HI and high HI after excluding the patients who experienced in-hospital ES episodes. Furthermore, the lagged effects of high HI on VTAs seem even longer (up to lag 3) in patients with ES. These results are shown in Supplementary Figure 1. Another sensitivity analysis excluding 10 patients with RV dysplasia and 13 patients with Brugada syndrome had similar outcomes.

## Discussion

Our data showed a significant seasonal effect in spring and summer among ICD patients with sustained VTAs, especially in patients with ES. In ES patients, monthly IR was highest in June and lowest in November. RH can modify the relationship between ambient temperature and the occurrence of VTAs in patients with ICD. A strong and modest nonlinear association between lower and higher HI, respectively, and sustained VTAs was found after adjusting for air pollutant concentrations. Patients with ICD had the lowest IR of sustained VTAs at a 24-h average HI of 24.5°C and had a higher IR either at a lower or higher HI. Moreover, lagged effects of HI on VTAs could only be demonstrated in patients with ES, which lasted longer for low HI (in the next 4 days) and were relatively shorter for high HI (in the next day only). Meanwhile, the lagged effects were more prominent than acute effects (lag 0) in patients with ES, especially for low HI. Furthermore, patients with ES were more vulnerable to heat stress than those without ES in both acute and lagged effects of high HI. To the best of our knowledge, this is the first study to evaluate the association between the occurrence of VTAs and apparent temperature in patients with an ICD living in a subtropical area.

Meteorological influences may activate pathophysiological mechanisms facilitating the occurrence of VTAs in susceptible patients. Cold weather conditions could contribute to the occurrence of arrhythmias through the activation of both the sympathetic nervous system and the coagulation system, which may reduce the ischemic threshold and in turn trigger VTA onset (17). Currently, several studies have examined the influence of temperature, mainly low temperature, on sustained VTAs. For every 1°C decrease in ambient temperature, the risk of ventricular arrhythmias is reportedly increased by 1.2% (18). Lower temperature and drier air are reportedly associated with an increased risk of ventricular arrhythmia onset among ICD patients (3). ICD shocks are reportedly 25% more common during extremely cold days and 8% more common during cold days (7). Despite the higher average minimum temperature in Taiwan, our study results were consistent with those of prior studies. The possible mechanism of sustained VTA occurrence during periods of thermal stress may be multifactorial (19). Increased skin blood flow and volume depletion due to sweating result in reduced coronary blood flow and a significant increase in blood viscosity. Heart rate and cardiac contractility are elevated during heat stress, which further worsens the myocardial oxygen supply–demand imbalance. Arterial thrombosis and disruptions in the autonomic nervous system may also pose a risk of VTAs (20). Moreover, extremely high temperature is associated with an increased risk of out-of-hospital cardiac arrest and cardiovascular death (10, 11). Most sudden deaths are caused by VTAs, which may occur even in individuals without a known cardiac disease (21). Studies in Korea and Japan found that VTA attack peaks during summer in patients with RV dysplasia, Brugada syndrome, and early repolarization syndrome (8, 9). In our study, we found that a higher HI is associated with a greater incidence of sustained VTAs, even after excluding 10 patients with arrhythmogenic RV dysplasia and 13 patients with Brugada syndrome. The different pathophysiology of cold and thermal stress may underlie the differential effects of HI on VTAs between patients with ischemic CVD and nonischemic CVD.

The association of cardiac arrhythmias with temperature is most likely influenced by the simultaneous effect of other atmospheric constituents. RH may also play a significant role in temperature-related arrhythmic effects, especially in the humid zones of places with subtropical and tropical climates. Low evaporation rate with high RH results in more heat retention in the body and heightens subjective hot sensation. Similarly, subjective cold sensation is heightened at a low temperature with high humidity. Fries et al. found a correlation of mean monthly felt temperature (including humidity) with frequency of VTAs (22), which is consistent with our results showing an association between HI and VTA incidence. Furthermore, using personal daily records, we have clarified the lag effects of low and high HI and the different associations in patients with ES and those without ES after adjusting for air pollutant concentrations. These lagged effects on VTAs are consistent with the effects of heat and cold on mortality (23). The lags for cold-related mortality were longer, and those for heat-related mortality shorter. In Taiwan, RH is high year-round, with an average of 76%. During the study period, the maximum temperature was 34°C, and the HI was augmented to 41°C because of high RH. By contrast, in countries like the UK, USA, Germany, and Switzerland, the RH is high in winter but is modest in summer (50–70%). This might account for the regional variations of study results related to the association between temperature and VTA.

ES is a state of electrical instability and is associated with high mortality. The cause of ES is a complex interaction between the autonomic nervous system, ischemia, and a predisposing electrophysiological substrate. However, data on ES predictors remain insufficient (24). ES incidence is reportedly not homogenous over time but seems to have a clustered pattern.

Significant adverse prognostic association of clustered VTAs is observable with even 2 VTA events within 3 months and increases with higher cluster density (25). An association between ES and an increase in monthly temperature variation had been reported (26). Our study is the first to show the association of HI with ES and the lagged effects of HI on VTA occurrence. Hence, in addition to cold weather, heat stress may play an important role in the incidence of ES. The combination effects of RH and temperature and the lagged effects of HI may partially explain the seasonal variation and clustered pattern of ES occurrence. However, the underlying mechanisms are unknown, and thus, further research is needed to clarify the interplay of heat stress and ES.

Recently, there has been a heightened awareness of the effects of extreme temperature due to global warming on health (24, 26), and such extreme temperature could also influence heat-related cardiac morbidity and mortality (11). However, relevant evidence in regions exposed to high HI, such as India and Southeast Asian countries where at least more than half of the world's population lives, remains limited. Our study showed a higher risk of sustained VTAs and ES with extremely high HI in patients with ICD in Taiwan, although the causality could not be confirmed because of the ecological study design. Nonetheless, precautions when dealing with extreme temperatures are recommended, and preparation for the reallocation of medical and social resources when facing climate changes would be vital in the near future.

The association between ambient air pollution and VTAs occurrence in patients with ICD remained inconclusive (27, 28). Our data showed no association between air pollutants, such as PM_2.5_ and PM_10_, and the incidence of sustained VTAs. This could be because the patients were advised to stay indoors during days with bad air quality, or they might have been at working places rather than at their residence when the incident occurred. Future studies are needed for the associations of VTAs with short-term air pollution exposure.

Several potential limitations should be noted in our study. First, we did not have the patients' individual meteorological exposure information. The study participants might have spent majority of their time indoors with air-conditioning during summer. Thus, nondifferential misclassification of exposure that likely attenuated the association toward the null for high HI was possible. The use of a heater in winter is not common in Taiwan; thus, the misclassification error for low HI is minimal in our study. Second, we did not have information on patients' socioeconomic status or occupation, which may have an effect on individual's VTA incidents. Nonetheless, the case-crossover analysis with patients serving as controls for themselves should have minimized the associated bias. Third, 87% of our study patients were secondary prevention ICD patients. Absence of uniform ICD programming in our patient population might affect the detection rate of VTAs. Most of ICD programming in our study was according to the characteristics of clinical VTAs and/or physician clinical experience. However, some slow VTs might be still und-detected in clinical scenario. Forth, patients who were lost-to-follow-up might lead to underestimate VTA episodes, which in turn could yield a bias on the study result as well. The case-crossover analysis with patients serving as controls for themselves could minimize this selection bias.

In conclusion, sustained VTAs of patients with ICD in Taiwan were strongly associated with low HI and modestly associated with high HI. Particularly, patients with ES were more vulnerable to heat stress than those non-ES patients, and the lagged effects of HI on VTAs could only be found in patients with ES. Avoiding exposure to low as well as high HI of those at risk of VTAs, especially those clustered VTAs, could improve the quality of life and may reduce mortality.

## Data Availability Statement

The raw data supporting the conclusions of this article will be made available by the authors, without undue reservation.

## Ethics Statement

The studies involving human participants were reviewed and approved by the study protocol was approved by the National Taiwan University Hospital Research Ethics Committee (study no. 201612133RINC). Written informed consent for participation was not required for this study in accordance with the national legislation and the institutional requirements.

## Author Contributions

H-CH, Y-BL, and C-CC designed the study. P-CS, J-SL, and CC were responsible for data collection. J-SL and C-CC were responsible for data acquisition and statistical analysis. H-CH, Y-BL, and C-CC were responsible for article writing. All authors contributed to the article and approved the submitted version.

## Conflict of Interest

The authors declare that the research was conducted in the absence of any commercial or financial relationships that could be construed as a potential conflict of interest.

## Supplementary Material

Supplementary Figure 1 Lag-specific percentage change in odds ratio per 1°C decrease (increase) when daily HI < 15°C (>30°C) for **(a)** all 172 patients, **(b)** 44 patients with ES, and **(c)** 128 non-ES patients.
