# Supplementary material for: Effects of Apparent Temperature on the Incidence of Ventricular Tachyarrhythmias in Patients With an Implantable Cardioverter–Defibrillator: Differential Association Between Patients With and Without Electrical Storm
Source: Front Med (Lausanne). 2021 Jan 15;7:624343. doi: 10.3389/fmed.2020.624343 (PMC7843936; doi:10.3389/fmed.2020.624343)

Supplementary file

Heat Index Formula

We calculated the HI (°F$)$ based on the following Steadman’s formula:

$$HI=0.5*\left\{ T+61.0+\left[ \left( T-68.0 \right)*1.2 \right]+\left( RH*0.094 \right) \right\}$$

if the calculated HI <80°F, where T is ambient temperature (°F) and RH is the relative humidity (%). If HI ≥80°F, then Rothfusz’s full formula is calculated as follows:

$HI=-42.379+2.049*T+10.143*RH-0.225*T*RH-0.0068*T^{2}-0.0548*{RH}^{2}+0.0012*T^{2}*RH+0.00085*T*{RH}^{2}+c_{9}T^{2}R^{2}$.

Adjustments were made by subtracting

$$\left[ \left( 13-RH \right)/4 \right]*SQRT\left\{ \left[ 17-ABS\left( T-95. \right) \right]/17 \right\}$$

if RH <13% and $80<T<112$ and adding $\left[ \left( RH-85 \right)/10 \right]*\left[ \left( 87-T \right)/5 \right]$

if RH >85% and $80<T<87$^22^. The calculated HI in degrees Fahrenheit (°F) was converted to degrees Celsius (°C).

Supplemental Figure

Lag-specific percentage change in odds ratio per 1℃ decrease (increase) when daily temperature <15℃ (>30℃) for (a) all 172 patients, (b) 45 patients with ES, and (c) 127 non-ES patients.


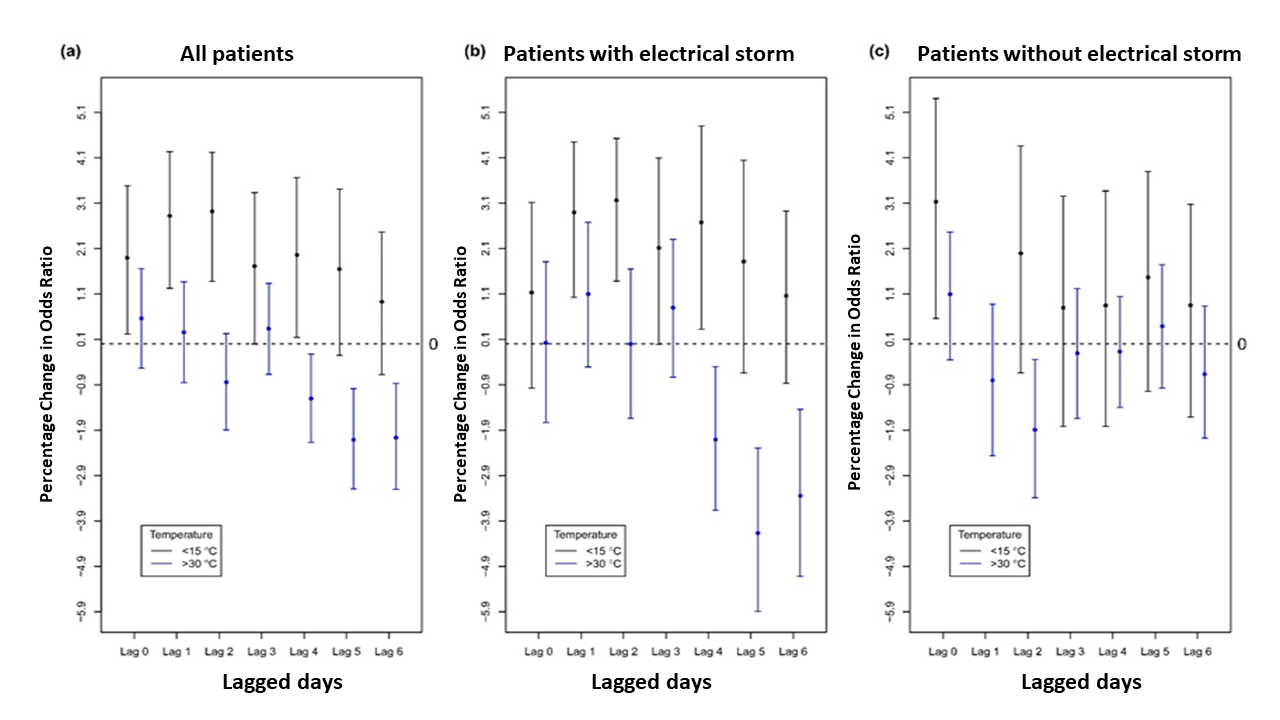

Supplement: Supplementary Figure 1 — Lag-specific percentage change in odds ratio per 1°C decrease (increase) when daily HI < 15°C (>30°C) for (a) all 172 patients, (b) 44 patients with ES, and (c) 128 non-ES patients. [file Table_1.DOCX]
